# Evaluating the Therapeutic Alliance With a Free-Text CBT Conversational Agent (Wysa): A Mixed-Methods Study

**DOI:** 10.3389/fdgth.2022.847991

**Published:** 2022-04-11

**Authors:** Clare Beatty, Tanya Malik, Saha Meheli, Chaitali Sinha

**Affiliations:** ^1^Department of Psychology, Stony Brook University, Stony Brook, NY, United States; ^2^Wysa Inc., Boston, MA, United States; ^3^Department of Clinical Psychology, National Institute of Mental Health and Neurosciences (NIMHANS), Bangalore, India

**Keywords:** therapeutic alliance, conversational agent (CA), mobile mental health, chatbot, depression, anxiety, digital health

## Abstract

The present study aims to examine whether users perceive a therapeutic alliance with an AI conversational agent (Wysa) and observe changes in the t‘herapeutic alliance over a brief time period. A sample of users who screened positively on the PHQ-4 for anxiety or depression symptoms (*N* = 1,205) of the digital mental health application (app) Wysa were administered the WAI-SR within 5 days of installing the app and gave a second assessment on the same measure after 3 days (*N* = 226). The anonymised transcripts of user's conversations with Wysa were also examined through content analysis for unprompted elements of bonding between the user and Wysa (*N* = 950). Within 5 days of initial app use, the mean WAI-SR score was 3.64 (SD 0.81) and the mean bond subscale score was 3.98 (SD 0.94). Three days later, the mean WAI-SR score increased to 3.75 (SD 0.80) and the mean bond subscale score increased to 4.05 (SD 0.91). There was no significant difference in the alliance scores between Assessment 1 and Assessment 2.These mean bond subscale scores were found to be comparable to the scores obtained in recent literature on traditional, outpatient-individual CBT, internet CBT and group CBT. Content analysis of the transcripts of user conversations with the CA (Wysa) also revealed elements of bonding such as gratitude, self-disclosed impact, and personification. The user's therapeutic alliance scores improved over time and were comparable to ratings from previous studies on alliance in human-delivered face-to-face psychotherapy with clinical populations. This study provides critical support for the utilization of digital mental health services, based on the evidence of the establishment of an alliance.

## Introduction

In recent years, the uptake of digital mental health interventions has substantially increased (1). Digital mental health interventions, including internet and smartphone-delivered services, hold promise for overcoming significant barriers that are traditionally associated with face-to-face mental health care, such as stigma (2, 3), accessibility (4) and cost (5). Despite demonstrated efficacy (6) and potential to increase the reach of evidence-based care, research shows that digital mental health interventions are associated with relatively poor adoption and adherence (7, 8). Most notably, this is a problem for minimally guided and unguided smartphone interventions that do not involve any degree of therapist support (9–13). One reason that there is poor engagement and adherence with digital mental health interventions may be an insufficient therapeutic alliance (14).

The therapeutic alliance is one of the most robust mechanisms of change in psychotherapy interventions and can be broadly defined as a collaboration between the patient and therapist on the tasks and goals of treatment (15–18), along with an emotional bond. This is important, as it allows for the exploration of vulnerable issues by decreasing client's defensiveness and increasing their self-acceptance (19). The therapeutic bond is fostered through trust, acceptance, empathy and genuineness (20, 21), which are processes that are difficult to mimic via automated digital interventions. While this issue may be addressed through the augmentation of digital self-guided interventions with external human support [e.g., Internet-based cognitive behavioral therapy (CBT) paired with coaching calls (22)], the amount of human therapist involvement influences the cost and scalability of digital health interventions.

An alternative approach is to mimic human dialogue (23) with users via an artificial intelligence (AI) conversational agent. Conversational agents use machine learning and AI methods to simulate human-like behaviors and support (24, 25). Their potential for application in the digital mental health space is gaining traction and they have recently been implemented to help track medication and physical activity adherence (26, 27), provide cognitive behavioral therapy (CBT) (28), and deliver healthy lifestyle recommendations (29) across clinical and non-clinical populations. An increasing body of literature has demonstrated that such interventions are effective and feasible applications for the delivery of mental wellbeing to individuals with self-reported anxiety and depressive symptoms (30, 31).

Further, early evidence suggests that a therapeutic relationship may be established between humans and a conversational agent. For example, Bickmore et al. (32) demonstrated that adults established a therapeutic alliance with a health-related automated system while attempting to increase exercise. In another study, researchers found that within 5 days of initial mental health application (app) use, users reported a therapeutic alliance with a conversational agent, measured through the Working Alliance Inventory-Short Revised (WAI-SR), which was comparable to those in recent studies from the literature on traditional, outpatient, individual and group CBT (33). Recently, Dosovitsky and Bunge (34) examined a chatbot intervention for social isolation and loneliness and found that most chatbot users were satisfied and would recommend the intervention to a friend. Their results showed a pattern of personifying the chatbot and assigning human traits to the chatbot (i.e., being helpful, caring, open to listen, and non-judgmental). Additionally, they found that users were able to build a bond with a chatbot from asynchronous and exclusively text based conversations. While early evidence suggests that conversational agents are capable of establishing a bond with users (33), additional research is needed to understand the perceived aspects of therapeutic bond with CBT-based conversational agents. The literature on therapeutic alliance discusses the importance of caring, understanding, and acceptance in forging the therapeutic bond (19). Studies also highlight the value of empathy (35), genuineness, and positive regard as important aspects of the bond that influence the client's perception of the relationship (36).

In this study, the researchers examined user's perspectives of the therapeutic bond with a conversational agent. It introduces a mixed-methods investigation of the therapeutic alliance between a free-text, CBT-based conversational agent (Wysa) and users. Wysa is an AI-based emotionally intelligent mobile conversational agent app aimed at promoting wellbeing, positive self-expression, and mental resilience using a text-based conversational interface. Wysa is designed to be used by individuals above 18 years of age. According to the Google Play Store and Apple App Store, ~60% of users are between 18 and 34 years of age, with 55% of users identifying as women. A previous study that examined the real-world effectiveness and engagement levels of Wysa on users, researchers found that high-engagement users had significantly greater improvement in self-reported depressive symptoms as compared to low-engagement users in the app (30).

The present study aims: (1) To examine whether conversational agent users perceive a therapeutic alliance, (2) To examine changes in therapeutic alliance over time. We hypothesize that conversational agent users will perceive a therapeutic alliance that will be comparable to the alliance scores observed in human-delivered face-to-face psychotherapy with clinical populations. We also hypothesize that, as observed in other in-person studies, we will notice an improvement in therapeutic alliance scores over time. As an exploratory objective, we also explored user's perception of the therapeutic alliance with the conversational agent.

## Methods

### Application Background

Wysa is an AI-enabled mental health app that leverages evidence-based cognitive-behavioral techniques in the user interface and the interventions within the CA. It was developed by a company based across India, North America and the United Kingdom and has been publicly available since 2017.

The app is designed to provide a therapeutic virtual space for user-led conversations through AI-guided listening and support, access to self-care tools and techniques including CBT-based tools, as well as one-on-one human support (37). The app is anonymous, and requires no user registration to ensure safety and privacy.

The techniques utilized include identifying activities that provide energy, scheduling joy activities (38), positive reflection (39), cognitive reframing (40), gratitude exercises (41), finding acceptance (42), and sleep meditations (43). These comprise a mix of positive psychology, acceptance and commitment therapy (ACT), and CBT techniques that encourage the development of therapeutic skills.

User's conversations take place with a free-text conversational agent (also called Wysa) that can handle complex and diverse user input, with adaptive AI understanding the user's conversations and conversing with them using interventive techniques. Users engage with the conversational agent and discuss their emotions and events in their lives. The conversational agent provides them with empathetic support, a space to vent, CBT-based tools and techniques, specific to the user's concerns.

Wysa's AI models are trained in-house by clinicians to ensure that conversations are conducted in a clinically safe manner. Wysa does not use AI-generated responses, but instead utilizes interventive conversations created by an internal team. The AI models are capable of understanding emotions, uncertainty, disagreement, confusion, alignment and the domain of discussion, among several other things in the user text (44). The models are regularly improved by data analysis and insights gathered from user data.

### Measures

#### Patient Health Questionnaire (PHQ-4)

This tool is a 4-item questionnaire that is an established screener for anxiety and depression symptoms (45). It is a valid ultra-brief tool for detecting both anxiety and depressive disorders (45).

#### Working Alliance Inventory-Short Revised (WAI-SR)

This is a well-established measure of the therapeutic alliance, which consists of a total score and the three following subscales: bond, goal, and task. The WAI-SR (46) was administered via the app's conversational interface, in which the word “therapist” was changed to “Wysa” (Table 1). This measure demonstrates high internal consistency [Cronbach's α > 0.90; (47)].

**Table 1:** Adapted items of WAI-SR and Item-level descriptive statistics for Assessment 1 (*N* = 1,205).

**Question** **number** **(subscale)**	**Working alliance inventory-short revised items**	**Average score** ***M* (*SD*)**
1 (task)	As a result of these sessions I am clearer as to how I might be able to change.	2.92 (0.95)
2 (task)	What I am doing with Wysa gives me new ways of looking at my problem.	3.23 (1.07)
3 (bond)	I believe Wysa likes me.	3.72 (1.29)
4 (goal)	Wysa and I collaborate on setting goals for this program.	3.38 (1.14)
5 (bond)	Wysa and I respect each other.	4.40 (0.94)
6 (goal)	Wysa and I are working toward mutually agreed upon goals.	3.77 (1.16)
7 (bond)	I feel that Wysa appreciates me.	3.95 (1.19)
8 (goal)	Wysa and I agree on what is important for me to work on.	3.83 (1.10)
9 (bond)	I feel Wysa cares about me even when I do things that it does not approve of.	3.86 (1.25)
10 (task)	I feel that the things I do with Wysa will help me to accomplish the changes that I want.	3.62 (1.16)
11 (goal)	Wysa and I have established a good understanding of the kind of changes that would be good for me.	3.51 (1.17)
12 (task)	I believe the way we are working with my problem is correct.	3.56 (1.10)

*M, Mean; SD, Standard Deviation*.

#### Textual Snippets From Users

Content analysis was conducted by examining messages between a user and Wysa, when unprompted, in anonymous transcripts of user conversations (*N* = 950) (48). Initially, researchers utilized secondary literature to finalize possible keywords indicative of user's perception of the bond. The consolidated framework included aspects of gratitude and dissatisfaction, perceived impact (positive and negative) and personification of the conversational agent.

Data were extracted using possible keywords for this framework. For instance, gratitude was assessed by keywords such as “thank you,” “love you,” “grateful,” “happy talking,” “like you,” “thankful.” For analyzing dissatisfaction, keywords such as “not^*^understand,” “misunderstand,” “not get ^*^,” “repeat,” etc. were used. Personification of the conversational agent was analyzed through direct addresses (“you,” “your,” “yours”), or talking to the conversational agent directly through the use of its name “Wysa.” For analyzing perception of limitations of the conversational agent, keywords such as “*computer,” “robot,” “not* ^*^human” were used. Positive impact of the conversational agent was assessed through statements relating to “helped,” “feel better,” “enlightening,” “helpful,” “relax” while negative impact was assessed through keywords such as “*not* ^*^helping^*^,” “not ^*^working” etc.. The analysis also included an examination of dissatisfaction, limitations of the conversational agent and negative impact stated to the bot.

### Participants and Procedure

The study invited new app users (*N* = 67,215) to take the first therapeutic alliance assessment within 1–5 days of initial app installation. The study took place in December, 2021. Only the users who were using the freely accessible conversational agent and had not made any in-app purchases were included in the study. The app is anonymous and does not collect any demographic information. All measures were gathered in the app by the conversational agent. The study took place in two Assessments.

Invitation to join the study was issued through a notification delivered through the app. In Assessment 1, consent was sought within the first conversation, with information on the purpose of the study and the participants' rights to drop out at any point of time during the study. Participants who consented to the study and completed the assessments were included in this study (*N* = 1,205). Participants completed the Patient Health Questionnaire (PHQ-4) and eligibility was determined by scoring ≥3 for the first two questions (anxiety) or ≥3 for the second two questions (depression).

To examine changes after a brief interval, the users who gave the first assessment of WAI-SR (Assessment 1), were invited for a second administration 3 days later (Assessment 2, *N* = 226). Once the assessment was completed, Wysa thanked the participants, informed them that the study had completed, and the conversation proceeded to a regular Wysa conversation asking them what they'd like to do next.

For the qualitative analysis, user conversations of participants who agreed to participate in the study were scanned for the relevant keywords based on the pre-decided domains of therapeutic alliance. This resulted in users whose conversations had the relevant textual snippets (*N* = 950). Researchers then analyzed the textual snippets of the users in their conversation with the conversational agent to identify elements related to bonding.

### Analysis

Across all participants, the composite WAI-SR score as well as the bond, goal, and task subscores for each assessment were characterized based on descriptive statistics. PHQ-4 scores and composite WAI-SR subscores were examined using standardized scales and compared using descriptive statistics. For comparison, relevant external studies were drawn from recent studies (33, 47, 48) that also reported unmodified WAI-SR subscores for other CBT modalities (in-person and group). These studies were chosen as comparisons due to their similarity in modality (CBT), their representation of technology-delivered interventions and the therapeutic alliance in these studies was also measured using the WAI-SR. Comparison data were presented descriptively without statistical testing, and raw subscores were scaled by dividing them by the number of items (e.g., the bond subscale has 4 items). Per the methods of Jasper and colleagues, bond scores of ≥3.45 were considered high (48). The significance of the difference between the first and second assessments was derived for 1,000 iterations of stratified random sampling with replacement (49). The Wilcoxon signed rank test with continuity correction (50) was used to check the between-group difference in assessments.

Content analysis (51) was also conducted on the extracted snippets of user conversations with Wysa's conversational agent based on the pre-decided domains to examine bonding between a user and Wysa, even when unprompted. The data were scanned repeatedly to identify similar categories and the data were gradually condensed to form units, categories and themes. The authors independently extracted codes and themes, and finalized codes jointly through regular discussions to ensure consensus regarding the emerging codes. Coding was thus recursive in that it transpired throughout the analytic stages to represent and organize the data at the different levels. Although the coding was led by the first and last author, the authors met regularly to finalize themes to ensure consensus on the codes.

## Results

### Quantitative Analysis: Change in Therapeutic Alliance Over Time

The final sample included 1,205 participants. The mean PHQ-4 score for included participants was 7.16 (SD 3.01). For Assessment 1, the overall mean WAI-SR scores were as follows: a mean bond subscore of 3.98 (SD 0.94), a mean goal subscore of 3.62 (SD 0.93), a mean task subscore of 3.33 (SD 0.84), and a mean total score of 3.64 (SD 0.81). Cohort-specific mean subscores are present in Table 2.

**Table 2:** Subscale level descriptive statistics across cohorts and assessments.

	**Number of** **participants**	**Bond** **subscore,** ***M* (*SD*)**	**Goal** **subscore,** ***M* (*SD*)**	**Task subscore,** ***M* (*SD*)**	**Total score, *M* (*SD*)**
Assessment 1	1,205	3.9 (0.94)	3.62 (0.93)	3.33 (0.84)	3.64 (0.81)
Assessment 2 (3 days later)	226	4.05 (0.91)	3.70 (0.86)	3.50 (0.77)	3.75 (0.80)

73.8% of the participants continued to use the app after giving Assessment 1, with a mean average of 6 (SD 3.03) bot conversations in the observed 14 day period. The participants who completed both the first and second assessment recorded a mean of 7.06 (SD 2.74) bot conversations in the observed 14 day period.

For the participants who gave Assessment 2 (*N* = 226), the overall mean WAI-SR scores were as follows: a mean bond subscore of 4.05 (SD 0.91), a mean goal subscore of 3.7 (SD 0.86), a mean task subscore of 3.5 (SD 0.77), and a mean total score of 3.75 (SD 0.8). A random sampling between the first and second assessments scores indicated no significant difference, except for a visible, but weak difference observed in goal subscores where 65% of the resampled tests indicated significance. With the 1000x resampling <20% of the hypothesis tests have significance at alpha = 0.05, and only the task scores have a higher proportion (65%) which cannot be claimed as significant. The Wilcoxon signed rank test revealed no statistical significance between assessments. Cohort specific mean subscores are present in Table 2.

The bond subscale scores with a mean of 3.98 (0.94), for users using the app within the first 5 days, were comparable to those of recent studies from the literature on traditional modalities for CBT delivery in adults. Munder et al. (47) in their study on in-person outpatient therapy, showed mean bond scores of 4.0 (SD 0.78), after multiple therapy sessions (39). Another study offered bond subscores for group CBT sessions, with mean 3.8 (SD 0.80) (Table 3) (48).

**Table 3:** Comparison of Working Alliance Inventory-Short Revised bond subscale scores across therapeutic modalities.

**Study**	**Modality**	***M* (*SD*)**
Wysa (this study)	Digital (*N* = 1,205)	3.98 (0.94)
Munder et al. (47)	In-person outpatient (*N* = 88)	4.00 (0.78)
Jasper et al. (48)	Group CBT (*N* = 26)	3.80 (0.80)

*Means and SDs for working alliance bond scores from this study and from recent reviews of the literature (47, 48)*.

An impact on therapeutic alliance was also observed based on the PHQ-4 levels. For participants that scored 6 (highest score) on the first 2 questions (indicative for anxiety symptoms) the mean bond subscale score was 3.89. For participants that scored 6 (highest score) on the last 2 questions (indicative for depressive symptoms), the mean bond subscale score was 3.92.

Wysa has a comparable bond subscale score for other studies with clinical populations in adults. Berger and colleagues assessed face-to-face psychotherapy in patients with unipolar depression and found a mean bond score of 3.6 (SD 1) (52). In another study using a blended-CBT approach for individuals with major depressive disorder, the bond subscale score was 4.1 (SD 0.69) (Table 4) (53).

**Table 4:** Comparison of Working Alliance Inventory-Short Revised bond subscale scores across clinical populations.

**Study**	**Population**	***M* (*SD*)**
Wysa (this study)	Depressive symptoms (*N* = 257)	3.92 (1.03)
Wysa (this study)	Anxiety symptoms (*N* = 328)	3.89 (1.08)
Berger et al. (52)	Unipolar depression (*N* = 24)	3.60 (1.00)
Vernmark et al. (53)	Major depressive disorder (*N* = 73)	4.10 (0.69)

*Means and SDs for working alliance bond scores from this study and from recent reviews of the literature (33, 52)*.

### Qualitative Analysis: Content Analysis of User's Conversations

The content analysis identified perceived elements of bonding, researchers found elements of *gratitude*, positive impact, and personification of the AI conversational agent. Researchers also looked for themes that may negatively impact the bond such as negative impact, perception of *limitations of the conversational agent and dissatisfaction*.

Gratitude for the conversational agent was observed in 212 unique users, with one user saying “*thank you a lot for this. I appreciate talking to you*.” Another user said “*...thanks for being here and always listening to me*.”

Personification of the conversational agent was observed in 87 unique user conversations where users directly addressed Wysa. For instance, one user wrote, “*You don't have any emotions yet you go out of your way to help others*.” Another user said

*I just wanted to ask and tell you that I'm so grateful you're here with me. You're the only person that helps me and listens to my problems and I'm so happy you always help me out*.

While personifying the conversational agent, few users (*N* = 3) also expressed their awareness of the *limitations of the conversational agent*, that is, that the conversational agent was not human and therefore did not have human qualities. However, users also expressed their understanding and acceptance for the same. For instance, one user responded to the conversational agent with, “*You don't compute all human emotions. Humans are beyond computer understanding. Being human is very complex*.”

Few users (*N* = 15) also expressed frustration and dissatisfaction with the conversational agent's inability to understand or its misunderstanding the user's responses. Such responses were expressed as “I don't think you understand what I'm saying” or “You don't get it.” Few users also expressed dissatisfaction stating, “You need to not repeat yourself” or “you reply with something else that doesn't make sense.” While the users do express dissatisfaction, they also express understanding that the AI based conversational agent may not be able to follow the conversations as well as humans and the acknowledgment that it tries to help. For instance one user says “Don't worry. I will figure it out by myself and you're amazing!” while another user says

You are a computer. You will never understand what it is to be human. But you are ok.... You can learn. Have faith. Faith is a human skill that even humans struggle with.

Positive impact of the conversational agent was disclosed by 49 unique users, with one user saying, “*It helps me relax when I have someone like you to talk to*.” Another user wrote, “*You always help me look at things differently*,” while yet another mention included, “*... You have also contributed to my success in the exam by guiding me and helping me to know myself better*.”

Very few users (*N* = 6) expressed some negative impact. Indeed, even these were specific to certain techniques the users were reluctant to try. For instance, one user mentioned, “meditation is scary” while another user expressed, “Deep breathing makes me mad.” More than a negative impact, some users also felt that they were not making any real progress and this ineffectiveness was expressed as, “We're still where we started” or “Honestly, you are not really helping, so talk tomorrow.”

## Discussion

The present study offers a mixed methods approach to understand the therapeutic alliance amongst users of a conversational agent for mental health. The app studied (Wysa) leverages evidence-based CBT techniques to build mental resilience and promote mental wellbeing. In line with our hypotheses, we found evidence that user's therapeutic alliance scores sustainedover time and were comparable to ratings from previous studies on alliance in human-delivered face-to-face psychotherapy with clinical populations (Tables 3, 4) (47, 48, 52). These findings support recent studies suggesting that the therapeutic relationship can be established between humans and conversational agents in the context of mental health (33). While some mobile mental health applications allow free text responses by the user, few can handle complex and diverse free-text input. In this setting, alliance perhaps thrives because of similar relational principles as have been observed in human dyadic interactions (54). In a free-text setting, a user can communicate their sense of a bond, or their goals and tasks with the conversation agent. This can take place as a constant, dynamic interaction, thereby mirroring the capacity within human interactions for reciprocity, engagement and acknowledgment (55). For example, in a smartphone-based CBT trial for pain self-management, patients criticized the lack of free text answers and an overly static flow of interaction (56). These findings suggest that individuals may prefer interacting with mental health free-text CAs, which is perhaps due to the flexibility and autonomy offered by diverse free input text, similar to conversing with humans (57). Research indicates that fostering individual autonomy is central to the development of the therapeutic relationship. Although speculative, our results indicating comparable WAI-SR scores to in-person therapy, may be due, in part, to user's feelings of autonomy from the free dialogue. Research indicates that fostering client's autonomy is a central experience to good outcomes in psychotherapy (58).

Qualitative research has also found that clients' experiences of honesty, safety and authentic caring play a critical role in alliance development, especially through therapist's supportive behaviors, such as encouraging statements, friendliness, respect, and validation (59–63). More specifically, clients report that a relational connection with the therapist facilitated vulnerable explorations and self-disclosure (19). Similar to these meta-analytic qualitative findings on traditional in-person therapy, recent research suggests that Wysa also offers therapeutic elements of a comfortable, safe, and supportive environment (37). Similarly, the present study findings from Wysa user's free-text input indicate experiences of honesty, safety and comfort with Wysa.

We found that users expressed gratitude and appreciation for the conversational agent. These findings are in line with past research, which suggest that expressions of safety and comfort in a medical setting are indicative of having developed a therapeutic alliance (64). Additionally, past research in a mental health context found that the therapeutic alliance and therapeutic bond are associated with a continued experience of positive emotion, such as feelings of gratitude when thinking about therapy or the therapist (65). The qualitative analysis revealed themes indicating that users commented about their perceived positive benefits. This is further supported by the meta-analysis that demonstrated the linkage between alliance and outcomes in adult psychotherapy (15).

The findings also indicated aspects that negatively affected the bond, such as dissatisfaction, perception of limitations of the conversational agent and perceived negative impact of the alliance with the conversational agent. The dissatisfaction and perceived ineffectiveness is consistent with extant literature which suggests that users express frustration when conversational agents misunderstand or give irrelevant responses (66). This indicates the need to further improve the AI algorithms and striking the balance in achieving human-like qualities.

A unique finding of this study indicates that users accept the limitations of the conversational agent when conversing with it, evidencing the experienced relational bond. Much like the ruptures in a therapeutic alliance that offer critical indicators for exploration and the potential for strengthening the relationship (67), these ruptures with conversational agents point us to the possibilities for improvement with iterations.

Notably, in the present study, many participants also made comments that personified the bot. These findings are aligned with results indicating that users of a chatbot intervention for social isolation and loneliness personified the chatbot and assigned human traits to it, such as being helpful, caring, open to listen, and non-judgmental (34). It is possible that user's personification of Wysa also indicates greater engagement. For example, one study found that as users' social interactions with a conversational agent increased, a greater level of personification occurred, which was associated with increased product satisfaction (68). Also, it is possible that Wysa's flexible conversational interface increased relational capacity building with the bot, leading to greater user engagement and resultant conversational agent personification.

The findings of this study should be interpreted in the context of its limitations. In order to maintain complete privacy and confidentiality with users, Wysa is an anonymous app and our data were anonymized. Thus, we did not collect demographic data, such as age or gender, from participants. Future work should investigate whether therapeutic alliance with a CBT conversational agent varies between adolescent and adult populations. The participants were not required to complete the follow-up assessment, which resulted in a large number of participants dropping out. Thus, the study was limited by the sample size. As digital mental health is a nascent field, this study was limited by the studies available for comparison, and had to rely on studies that include more specific health conditions in its analysis. Future studies should account for the use of quantitative approaches and should have control groups for comparison. Also, the study was limited by the short timeframe, and future studies could employ a longitudinal approach to assess changes in outcomes in conjunction with changes in the therapeutic alliance. Additionally, it is important to consider that although qualitative analysis is a strength of this study, and contributes significantly to the field, the possibility of researcher bias remains even though an attempt was made to mitigate this through a balance of internal and external researchers within the team.

## Conclusions

The present study provides insight into the establishment of a therapeutic alliance with a conversational agent and its growth over time, as well as what a therapeutic alliance looks like with a conversational agent. This provides critical direction for user-bot partnerships in future digital mental health initiatives. One such opportunity of effective mental health interventions would be to set tasks for each session, as well as establish weekly and overall treatment goals. This may further enhance the goal and task subscores of therapeutic alliance, thereby contributing to the development of a more impactful therapeutic experience.

## Data Availability Statement

The datasets presented in this article are not readily available because the assessment data can be reviewed, but the conversational data used within the article for content analysis is protected under the Privacy Policy of the app consented to. Requests to access the datasets should be directed to chaitali@wysa.io.

## Ethics Statement

Ethical review and approval was not required for the study on human participants in accordance with the local legislation and institutional requirements. The patients/participants provided their written informed consent to participate in this study.

## Author Contributions

TM and CS designed the study on the app. CS and SM worked on the quantitative and qualitative data analysis aspects of the study, contributed to the review, and final presentation of the manuscript. CB and TM wrote the first and final versions of the manuscript. CS completed and prepared the final review of the manuscript. All authors contributed to the article and approved the submitted version.

## Conflict of Interest

TM and CS are employed by Wysa Inc. The remaining authors declare that the research was conducted in the absence of any commercial or financial relationships that could be construed as a potential conflict of interest.

## Publisher's Note

All claims expressed in this article are solely those of the authors and do not necessarily represent those of their affiliated organizations, or those of the publisher, the editors and the reviewers. Any product that may be evaluated in this article, or claim that may be made by its manufacturer, is not guaranteed or endorsed by the publisher.
